# Crystal structure of 3,5-bis­(4-chloro­phen­yl)-1-propyl-1,3,5-tri­aza­cyclo­hexane

**DOI:** 10.1107/S1600536814019060

**Published:** 2014-08-30

**Authors:** Leila Lefrada, Ahcene Bouchemma, Sofiane Bouacida, Nicolas Claiser, Mohamed Souhassou

**Affiliations:** a Laboratoire de chimie appliquée et technologie des matériaux, LCATM, Université Oum El Bouaghi, Algeria; b Département Sciences de la matière, Faculté des sciences exactes et sciences de la nature et de la vie, Université Oum El Bouaghi, Algeria; c Unité de recherche de CHimie de l’environnement et Moléculaire Structurale, CHEMS, Faculté des sciences exactes, Université Constantine 1, Algeria; d Laboratoire de Cristallographie, Résonance Magnétique et Modélisations, UMR 7036 CNRS, Institut Jean Barriol, Université de Lorraine, Nancy, France

**Keywords:** crystal structure, tri­aza­cyclo­hexa­ne, C—H⋯π inter­actions

## Abstract

In the title mol­ecule, C_18_H_21_Cl_2_N_3_, the tri­aza­cyclo­hexane ring adopts a chair conformation with both 4-chloro­phenyl substituents in axial positions and the propyl group in an equatorial site. The dihedral angle between the planes of the benzene rings is 49.5 (1)°. In the crystal, mol­ecules are arranged in a head-to-tail fashion, forming columns along [010], and pairs of weak C—H⋯π inter­actions form inversion dimers between columns.

## Related literature   

For conformations of 1,3,5-triaryl derivatives of 1,3,5-tri­aza­cyclo­hexane, see: Baker *et al.* (1978 ▶); Bouchemma *et al.* (1989 ▶, 1990 ▶); Bushweller (1995 ▶); Kleinpeter *et al.* (2005 ▶); Duke *et al.* (1973 ▶); Gilardi *et al.* (2003 ▶); Giumanini *et al.* (1985 ▶); Latreche *et al.* (2006 ▶); Mloston *et al.* (2006 ▶); Freeman *et al.* (2005 ▶); Wiberg *et al.* (1999 ▶).
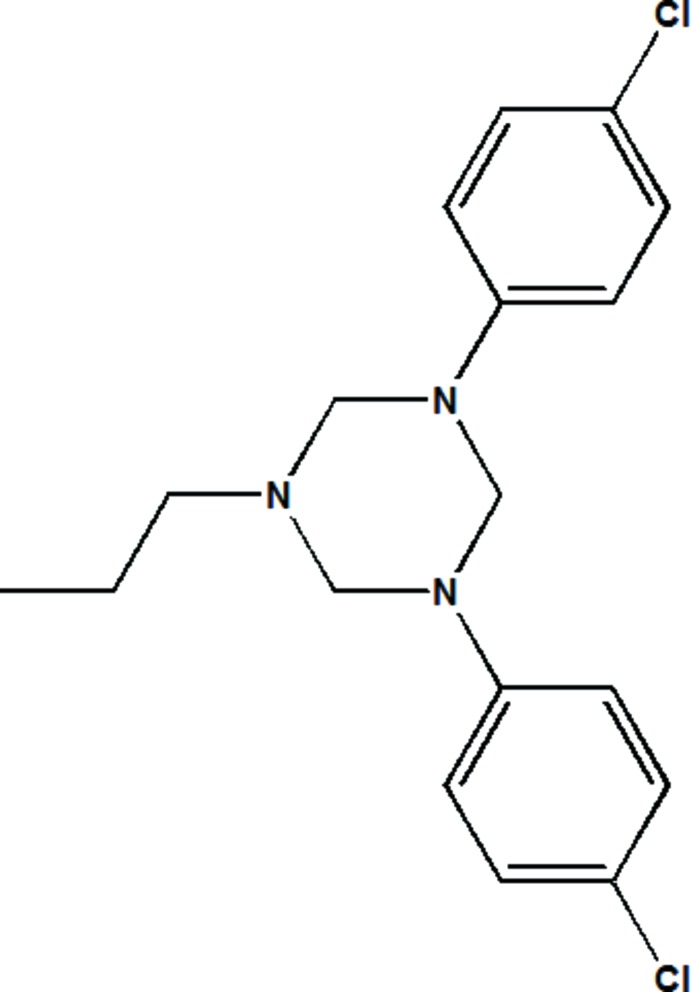


## Experimental   

### Crystal data  

C_18_H_21_Cl_2_N_3_
*M* *_r_* = 350.28
Triclinic, 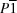

*a* = 6.0785 (3) Å
*b* = 10.3190 (6) Å
*c* = 14.4360 (8) Å
α = 91.570 (3)°
β = 91.946 (2)°
γ = 99.055 (3)°
*V* = 893.19 (8) Å^3^
*Z* = 2
Mo *K*α radiation
μ = 0.37 mm^−1^
*T* = 295 K
0.22 × 0.13 × 0.07 mm

### Data collection  

Nonius KappaCCD diffractometer
Absorption correction: multi-scan (Blessing, 1995 ▶) *T*_min_ = 0.274, *T*_max_ = 0.467
13643 measured reflections
3448 independent reflections
2751 reflections with *I* > 2σ(*I*)
*R*_int_ = 0.016

### Refinement  

*R*[*F* ^2^ > 2σ(*F* ^2^)] = 0.049
*wR*(*F* ^2^) = 0.142
*S* = 1.03
3448 reflections
209 parameters
H-atom parameters constrained
Δρ_max_ = 0.45 e Å^−3^
Δρ_min_ = −0.25 e Å^−3^

### 

Data collection: *COLLECT* (Nonius, 1997 ▶); cell refinement: *SCALEPACK* (Otwinowski & Minor, 1997 ▶); data reduction: *DENZO* (Otwinowski & Minor, 1997 ▶) and *SCALEPACK*; program(s) used to solve structure: *SIR2002* (Burla *et al.*, 2005 ▶); program(s) used to refine structure: *SHELXL97* (Sheldrick, 2008 ▶); molecular graphics: *ORTEP-3 for Windows* (Farrugia, 2012 ▶) and *DIAMOND* (Brandenburg & Berndt, 2001 ▶); software used to prepare material for publication: *WinGX* (Farrugia, 2012 ▶).

## Supplementary Material

Crystal structure: contains datablock(s) I. DOI: 10.1107/S1600536814019060/lh5723sup1.cif

Structure factors: contains datablock(s) I. DOI: 10.1107/S1600536814019060/lh5723Isup2.hkl

Supporting information file. DOI: 10.1107/S1600536814019060/lh5723Isup3.cml

**Fig. 1.**
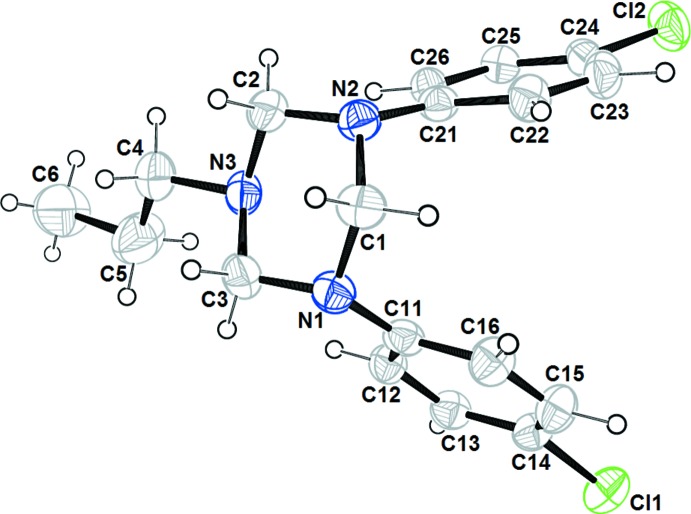
The molecular structure of the title compound with displacement ellipsoids drawn at the 50% probability level.

. DOI: 10.1107/S1600536814019060/lh5723fig1.tif
The mol­ecular structure of the title compound with displacement ellipsoids drawn at the 50% probability level.

**Fig. 2.**
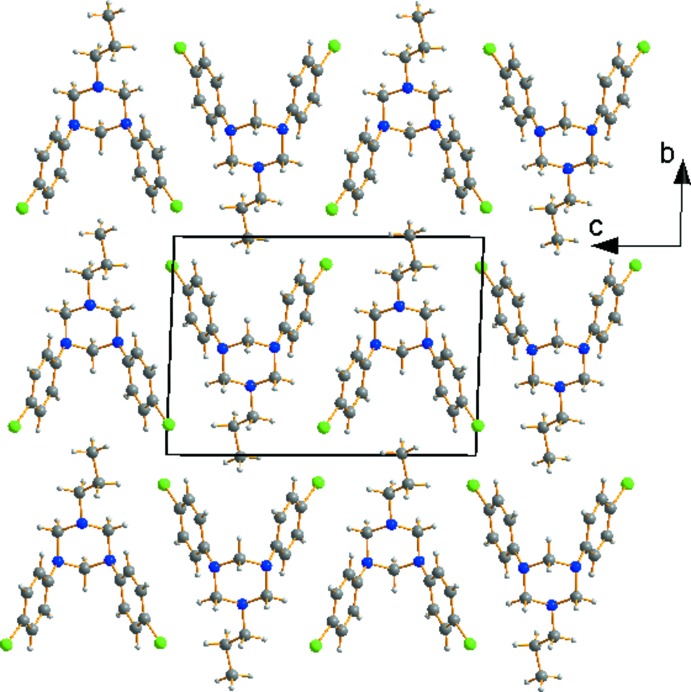
Part of the crystal structure of the title compound showing the 'head to tail' arrangement of molecules arranged in columns.

. DOI: 10.1107/S1600536814019060/lh5723fig2.tif
Part of the crystal structure of the title compound showing the ’head to tail’ arrangement of mol­ecules arranged in columns.

CCDC reference: 1020727

Additional supporting information:  crystallographic information; 3D view; checkCIF report

## Figures and Tables

**Table 1:** Hydrogen-bond geometry (Å, °) *Cg* is centroid of C21–C26 ring.

*D*—H⋯*A*	*D*—H	H⋯*A*	*D*⋯*A*	*D*—H⋯*A*
C2—H2*A*⋯*Cg* ^i^	0.96	2.92	3.668 (3)	134

Symmetry code: (i) 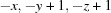.

## supplementary crystallographic information

### S1. Comment

The conformational behaviour of substituted cyclohexanes as well as heterocyclohexanes has been the subject of numerous studies (Bushweller, 1995). The ring normally adopts the chair conformation unless specific intramolecular interactions stabilize the twist (Kleinpeter *et al.*, 2005) or boat conformers (Freeman *et al.*, 2005). Saturated six-membered rings are prevalent in organic chemistry. For cyclohexane, experimental and computational studies have established that the chair conformation is 5.5 kcal/ mol more stable than the twist form (Wiberg *et al.*, 1999). N,*N*',*N*''-Trisubstituted 1,3,5-triazinanes are of interest as precursors for the preparation of different N-substituted imidazoles (Mloston *et al.*, 2006). The heterocyclic nucleus is expected to adopt a chair conformation and four distinct patterns of substituent orientation have to be considered, eee, eea, eaa and aaa, where e = equatorial and a = axial, with each of the conformers having axial repulsions involving the substituents or lone pairs of electrons on the N atoms. Several 1,3,5-trialkyl derivatives have been investigated in solution by dipole moment measurements and the results interpreted in terms of the eee conformer, the eea conformer (Baker *et al.*, 1978), and varying amounts of the eee, eea and eaa conformers (Duke *et al.*, 1973). Various 1,3,5-triaryl-1,3,5-triazacyclohexanes adopt the diaxial-equatorial orientation of substituents in the solid state thus avoiding 1,3-diaxial lone-pair repulsions (Giumanini *et al.* 1985; Gilardi *et al.* 2003 Bouchemma *et al.* 1989; 1990).

In the present work, a new derivate (I) of triazacyclohexane is reported and molecular structure is shown in Fig. 1. The 1,3,5-triazacycolohexane ring is in a chair conformation which is typical of this ring (Gilardi *et al.* 2003). The stucture of a similar compound *viz* 1-propyl-3-5-bis-(4-fluorophenyl)-1,3,5- triazacycolohexane (II) has been reported (Latreche *et al.* 2006). In both (I) and (II) the heterocyclic rings adopt chair conformations with two fluorophenyl substituents situated in axial positions and a third group (propyl) equatorial. The dihedral angle between the benzene rings (C11-C16/C21-C26) is 49.5 (1)°. In the crystal, molecules are arranged in a 'head to tail' fashion forming columns along [010] (see Fig. 2) and pairs weak C—H···π interactions form inversion dimers between columns.

### S2. Experimental

The title compound was obtained by mixing a 2:1:1 stoichiometric ratio of propylamine and 4-chloroaniline with formalin in ethanol (25 ml) at 293K. The resulting solution was evaporated on a rotary evaporator to dryness and the white residue was recrystallized from cyclohexane.

### S3. Refinement

All non-H atoms were refined with anisotropic atomic displacement parameters. All H atoms were located in differnce Fourier maps but introduced in calculated positions and treated as riding on their parent C atom, with C—H distances of 0.93 Å (C_aromatic_), 0.97 Å (C_methylene_) and 0.96 Å (C_methyl_) with *U*_iso_(H) = 1.2 *U*_eq_(C_aromatic_ and C_methylene_) or 1.5 *U*_eq_(C_methyl_).

### Crystal data

**Table:**

C_18_H_21_Cl_2_N_3_	*Z* = 2
*M**_r_* = 350.28	*F*(000) = 368
Triclinic, *P*1	*D*_x_ = 1.302 Mg m^−^^3^
Hall symbol: -P 1	Mo *K*α radiation, λ = 0.71073 Å
*a* = 6.0785 (3) Å	Cell parameters from 13346 reflections
*b* = 10.3190 (6) Å	θ = 1.5–27.3°
*c* = 14.4360 (8) Å	µ = 0.37 mm^−^^1^
α = 91.570 (3)°	*T* = 295 K
β = 91.946 (2)°	Prism, colourless
γ = 99.055 (3)°	0.22 × 0.13 × 0.07 mm
*V* = 893.19 (8) Å^3^	

### Data collection

**Table:**

Nonius KappaCCD diffractometer	2751 reflections with *I* > 2σ(*I*)
Graphite monochromator	*R*_int_ = 0.016
ω and φ scans	θ_max_ = 27.3°, θ_min_ = 1.4°
Absorption correction: multi-scan (Blessing, 1995)	*h* = −7→7
*T*_min_ = 0.274, *T*_max_ = 0.467	*k* = −12→12
13643 measured reflections	*l* = −17→17
3448 independent reflections	

### Refinement

**Table:**

Refinement on *F*^2^	Primary atom site location: structure-invariant direct methods
Least-squares matrix: full	Secondary atom site location: difference Fourier map
*R*[*F*^2^ > 2σ(*F*^2^)] = 0.049	Hydrogen site location: inferred from neighbouring sites
*wR*(*F*^2^) = 0.142	H-atom parameters constrained
*S* = 1.03	*w* = 1/[σ^2^(*F*_o_^2^) + (0.0652*P*)^2^ + 0.413*P*] where *P* = (*F*_o_^2^ + 2*F*_c_^2^)/3
3448 reflections	(Δ/σ)_max_ < 0.001
209 parameters	Δρ_max_ = 0.45 e Å^−^^3^
0 restraints	Δρ_min_ = −0.25 e Å^−^^3^

### Special details

**Table:**

Geometry. All e.s.d.'s (except the e.s.d. in the dihedral angle between two l.s. planes) are estimated using the full covariance matrix. The cell e.s.d.'s are taken into account individually in the estimation of e.s.d.'s in distances, angles and torsion angles; correlations between e.s.d.'s in cell parameters are only used when they are defined by crystal symmetry. An approximate (isotropic) treatment of cell e.s.d.'s is used for estimating e.s.d.'s involving l.s. planes.
Refinement. Refinement of *F*^2^ against ALL reflections. The weighted *R*-factor *wR* and goodness of fit *S* are based on *F*^2^, conventional *R*-factors *R* are based on *F*, with *F* set to zero for negative *F*^2^. The threshold expression of *F*^2^ > σ(*F*^2^) is used only for calculating *R*-factors(gt) *etc*. and is not relevant to the choice of reflections for refinement. *R*-factors based on *F*^2^ are statistically about twice as large as those based on *F*, and *R*- factors based on ALL data will be even larger.

### Fractional atomic coordinates and isotropic or equivalent isotropic displacement parameters (Å^2^)

**Table:**

	*x*	*y*	*z*	*U*_iso_*/*U*_eq_	
C1	−0.3001 (4)	0.4766 (3)	0.25020 (16)	0.0613 (6)	
H1A	−0.3547	0.383	0.2459	0.074*	
H1B	−0.4281	0.5221	0.2505	0.074*	
C2	−0.0783 (4)	0.6456 (2)	0.34207 (17)	0.0633 (6)	
H2A	0.0103	0.6653	0.3993	0.076*	
H2B	−0.1997	0.6964	0.3432	0.076*	
C3	−0.0772 (5)	0.6525 (2)	0.17830 (18)	0.0652 (6)	
H3A	−0.1979	0.7039	0.1778	0.078*	
H3B	0.013	0.6767	0.1254	0.078*	
C4	0.1485 (6)	0.8253 (3)	0.2756 (2)	0.0847 (8)	
H4A	0.0283	0.8751	0.2638	0.102*	
H4B	0.2019	0.8438	0.3394	0.102*	
C5	0.3281 (8)	0.8689 (4)	0.2147 (3)	0.1185 (14)	
H5A	0.2688	0.865	0.1513	0.142*	
H5B	0.4368	0.8096	0.2185	0.142*	
C6	0.4450 (8)	1.0093 (4)	0.2390 (3)	0.1321 (17)	
H6A	0.3366	1.0677	0.2395	0.198*	
H6B	0.5531	1.0364	0.1935	0.198*	
H6C	0.5184	1.0117	0.2991	0.198*	
C11	−0.0360 (4)	0.4255 (2)	0.13332 (14)	0.0505 (5)	
C12	0.1801 (4)	0.4642 (2)	0.10651 (16)	0.0556 (5)	
H12	0.2467	0.5511	0.1166	0.067*	
C13	0.2992 (4)	0.3762 (2)	0.06492 (16)	0.0590 (6)	
H13	0.4436	0.4041	0.0465	0.071*	
C14	0.2027 (4)	0.2473 (2)	0.05101 (16)	0.0585 (6)	
C15	−0.0100 (4)	0.2056 (2)	0.07759 (19)	0.0691 (7)	
H15	−0.0742	0.1181	0.0685	0.083*	
C16	−0.1281 (4)	0.2940 (2)	0.11776 (19)	0.0666 (6)	
H16	−0.2732	0.2653	0.135	0.08*	
C21	−0.0324 (4)	0.4175 (2)	0.36755 (14)	0.0491 (5)	
C22	−0.1257 (4)	0.2865 (2)	0.37598 (18)	0.0622 (6)	
H22	−0.2735	0.2589	0.3566	0.075*	
C23	−0.0036 (4)	0.1967 (2)	0.41250 (19)	0.0660 (6)	
H23	−0.0682	0.1094	0.4173	0.079*	
C24	0.2138 (4)	0.2375 (2)	0.44168 (16)	0.0583 (6)	
C25	0.3124 (4)	0.3650 (2)	0.43325 (16)	0.0565 (5)	
H25	0.4605	0.3913	0.4526	0.068*	
C26	0.1900 (4)	0.4544 (2)	0.39570 (15)	0.0536 (5)	
H26	0.2579	0.5408	0.3892	0.064*	
N1	−0.1697 (3)	0.51418 (19)	0.16948 (13)	0.0578 (5)	
N2	−0.1687 (3)	0.50789 (19)	0.33683 (13)	0.0561 (5)	
N3	0.0600 (4)	0.68403 (18)	0.26379 (14)	0.0615 (5)	
Cl1	0.34990 (13)	0.13680 (7)	−0.00400 (5)	0.0841 (3)	
Cl2	0.36408 (14)	0.12627 (7)	0.49405 (6)	0.0865 (3)	

### Atomic displacement parameters (Å^2^)

**Table:**

	*U*^11^	*U*^22^	*U*^33^	*U*^12^	*U*^13^	*U*^23^
C1	0.0497 (13)	0.0749 (16)	0.0606 (14)	0.0138 (11)	0.0016 (10)	0.0033 (11)
C2	0.0733 (16)	0.0592 (14)	0.0614 (14)	0.0235 (12)	0.0035 (12)	−0.0017 (11)
C3	0.0750 (16)	0.0586 (14)	0.0659 (15)	0.0211 (12)	0.0029 (12)	0.0117 (11)
C4	0.108 (2)	0.0557 (15)	0.090 (2)	0.0095 (15)	0.0082 (18)	0.0044 (14)
C5	0.146 (4)	0.088 (2)	0.115 (3)	−0.010 (2)	0.040 (3)	−0.009 (2)
C6	0.173 (4)	0.088 (3)	0.115 (3)	−0.044 (3)	0.017 (3)	0.006 (2)
C11	0.0516 (12)	0.0559 (12)	0.0421 (10)	0.0039 (10)	−0.0054 (9)	0.0039 (9)
C12	0.0574 (13)	0.0499 (12)	0.0562 (12)	−0.0013 (10)	−0.0019 (10)	0.0039 (10)
C13	0.0545 (13)	0.0635 (14)	0.0572 (13)	0.0030 (11)	0.0037 (10)	0.0052 (11)
C14	0.0639 (14)	0.0586 (13)	0.0519 (12)	0.0084 (11)	−0.0040 (10)	−0.0039 (10)
C15	0.0691 (16)	0.0530 (14)	0.0799 (17)	−0.0045 (12)	−0.0021 (13)	−0.0076 (12)
C16	0.0536 (13)	0.0641 (15)	0.0778 (16)	−0.0036 (11)	0.0046 (12)	−0.0022 (12)
C21	0.0510 (12)	0.0533 (12)	0.0431 (11)	0.0077 (9)	0.0077 (9)	−0.0002 (9)
C22	0.0524 (13)	0.0587 (14)	0.0725 (15)	−0.0012 (11)	0.0051 (11)	0.0020 (11)
C23	0.0690 (16)	0.0492 (13)	0.0782 (16)	0.0015 (11)	0.0125 (13)	0.0071 (11)
C24	0.0664 (15)	0.0554 (13)	0.0564 (13)	0.0164 (11)	0.0126 (11)	0.0087 (10)
C25	0.0528 (12)	0.0610 (13)	0.0561 (13)	0.0102 (10)	0.0020 (10)	0.0024 (10)
C26	0.0562 (13)	0.0486 (12)	0.0541 (12)	0.0018 (10)	0.0059 (10)	0.0023 (9)
N1	0.0583 (11)	0.0619 (11)	0.0538 (11)	0.0113 (9)	−0.0016 (8)	0.0046 (9)
N2	0.0567 (11)	0.0603 (11)	0.0525 (10)	0.0130 (9)	0.0024 (8)	0.0000 (8)
N3	0.0734 (13)	0.0446 (10)	0.0665 (12)	0.0104 (9)	−0.0022 (10)	0.0023 (9)
Cl1	0.0883 (5)	0.0780 (5)	0.0872 (5)	0.0207 (4)	0.0049 (4)	−0.0199 (4)
Cl2	0.0940 (5)	0.0765 (5)	0.0981 (6)	0.0357 (4)	0.0131 (4)	0.0278 (4)

### Geometric parameters (Å, º)

**Table:**

C1—N1	1.458 (3)	C11—C16	1.393 (3)
C1—N2	1.461 (3)	C11—N1	1.417 (3)
C1—H1A	0.97	C12—C13	1.384 (3)
C1—H1B	0.97	C12—H12	0.93
C2—N2	1.439 (3)	C13—C14	1.372 (3)
C2—N3	1.458 (3)	C13—H13	0.93
C2—H2A	0.97	C14—C15	1.368 (4)
C2—H2B	0.97	C14—Cl1	1.747 (2)
C3—N1	1.450 (3)	C15—C16	1.374 (4)
C3—N3	1.465 (3)	C15—H15	0.93
C3—H3A	0.97	C16—H16	0.93
C3—H3B	0.97	C21—C26	1.389 (3)
C4—C5	1.448 (5)	C21—C22	1.392 (3)
C4—N3	1.475 (3)	C21—N2	1.412 (3)
C4—H4A	0.97	C22—C23	1.380 (4)
C4—H4B	0.97	C22—H22	0.93
C5—C6	1.536 (4)	C23—C24	1.371 (4)
C5—H5A	0.97	C23—H23	0.93
C5—H5B	0.97	C24—C25	1.367 (3)
C6—H6A	0.96	C24—Cl2	1.747 (2)
C6—H6B	0.96	C25—C26	1.384 (3)
C6—H6C	0.96	C25—H25	0.93
C11—C12	1.383 (3)	C26—H26	0.93
			
N1—C1—N2	111.86 (19)	C13—C12—H12	119.3
N1—C1—H1A	109.2	C14—C13—C12	119.6 (2)
N2—C1—H1A	109.2	C14—C13—H13	120.2
N1—C1—H1B	109.2	C12—C13—H13	120.2
N2—C1—H1B	109.2	C15—C14—C13	120.5 (2)
H1A—C1—H1B	107.9	C15—C14—Cl1	119.76 (19)
N2—C2—N3	111.81 (18)	C13—C14—Cl1	119.70 (19)
N2—C2—H2A	109.3	C14—C15—C16	119.6 (2)
N3—C2—H2A	109.3	C14—C15—H15	120.2
N2—C2—H2B	109.3	C16—C15—H15	120.2
N3—C2—H2B	109.3	C15—C16—C11	121.7 (2)
H2A—C2—H2B	107.9	C15—C16—H16	119.1
N1—C3—N3	112.04 (19)	C11—C16—H16	119.1
N1—C3—H3A	109.2	C26—C21—C22	117.6 (2)
N3—C3—H3A	109.2	C26—C21—N2	123.0 (2)
N1—C3—H3B	109.2	C22—C21—N2	119.3 (2)
N3—C3—H3B	109.2	C23—C22—C21	121.4 (2)
H3A—C3—H3B	107.9	C23—C22—H22	119.3
C5—C4—N3	113.4 (3)	C21—C22—H22	119.3
C5—C4—H4A	108.9	C24—C23—C22	119.4 (2)
N3—C4—H4A	108.9	C24—C23—H23	120.3
C5—C4—H4B	108.9	C22—C23—H23	120.3
N3—C4—H4B	108.9	C25—C24—C23	120.9 (2)
H4A—C4—H4B	107.7	C25—C24—Cl2	119.54 (19)
C4—C5—C6	112.8 (3)	C23—C24—Cl2	119.48 (19)
C4—C5—H5A	109	C24—C25—C26	119.5 (2)
C6—C5—H5A	109	C24—C25—H25	120.2
C4—C5—H5B	109	C26—C25—H25	120.2
C6—C5—H5B	109	C25—C26—C21	121.2 (2)
H5A—C5—H5B	107.8	C25—C26—H26	119.4
C5—C6—H6A	109.5	C21—C26—H26	119.4
C5—C6—H6B	109.5	C11—N1—C3	118.73 (19)
H6A—C6—H6B	109.5	C11—N1—C1	118.44 (19)
C5—C6—H6C	109.5	C3—N1—C1	109.38 (19)
H6A—C6—H6C	109.5	C21—N2—C2	118.41 (19)
H6B—C6—H6C	109.5	C21—N2—C1	118.07 (18)
C12—C11—C16	117.3 (2)	C2—N2—C1	109.76 (19)
C12—C11—N1	123.2 (2)	C2—N3—C3	108.2 (2)
C16—C11—N1	119.4 (2)	C2—N3—C4	108.2 (2)
C11—C12—C13	121.3 (2)	C3—N3—C4	112.7 (2)
C11—C12—H12	119.3		
			
N3—C4—C5—C6	−170.1 (3)	C16—C11—N1—C3	−175.1 (2)
C16—C11—C12—C13	0.7 (3)	C12—C11—N1—C1	−136.1 (2)
N1—C11—C12—C13	−175.0 (2)	C16—C11—N1—C1	48.3 (3)
C11—C12—C13—C14	−0.9 (3)	N3—C3—N1—C11	−83.0 (3)
C12—C13—C14—C15	0.2 (4)	N3—C3—N1—C1	57.2 (3)
C12—C13—C14—Cl1	178.45 (18)	N2—C1—N1—C11	84.7 (2)
C13—C14—C15—C16	0.6 (4)	N2—C1—N1—C3	−55.7 (3)
Cl1—C14—C15—C16	−177.6 (2)	C26—C21—N2—C2	−5.2 (3)
C14—C15—C16—C11	−0.8 (4)	C22—C21—N2—C2	170.6 (2)
C12—C11—C16—C15	0.1 (4)	C26—C21—N2—C1	131.2 (2)
N1—C11—C16—C15	176.0 (2)	C22—C21—N2—C1	−53.0 (3)
C26—C21—C22—C23	1.2 (3)	N3—C2—N2—C21	81.6 (2)
N2—C21—C22—C23	−174.9 (2)	N3—C2—N2—C1	−58.1 (3)
C21—C22—C23—C24	0.4 (4)	N1—C1—N2—C21	−83.5 (3)
C22—C23—C24—C25	−1.4 (4)	N1—C1—N2—C2	56.4 (3)
C22—C23—C24—Cl2	176.34 (19)	N2—C2—N3—C3	58.6 (2)
C23—C24—C25—C26	0.7 (4)	N2—C2—N3—C4	−179.0 (2)
Cl2—C24—C25—C26	−177.02 (17)	N1—C3—N3—C2	−58.3 (3)
C24—C25—C26—C21	0.9 (3)	N1—C3—N3—C4	−177.8 (2)
C22—C21—C26—C25	−1.8 (3)	C5—C4—N3—C2	166.4 (3)
N2—C21—C26—C25	174.05 (19)	C5—C4—N3—C3	−74.0 (4)
C12—C11—N1—C3	0.5 (3)		

### Hydrogen-bond geometry (Å, º)

**Table:**

*D*—H···*A*	*D*—H	H···*A*	*D*···*A*	*D*—H···*A*
C2—H2*A*···*Cg*^i^	0.96	2.92	3.668 (3)	134

Symmetry code: (i) −*x*, −*y*+1, −*z*+1.
