# Supplementary figures and images for: Crystal structure of 3,5-bis­(4-chloro­phen­yl)-1-propyl-1,3,5-tri­aza­cyclo­hexane
Source: Acta Crystallogr Sect E Struct Rep Online. 2014 Aug 30;70(Pt 9):o1061–2. doi: 10.1107/S1600536814019060 (PMC4186157; doi:10.1107/S1600536814019060)

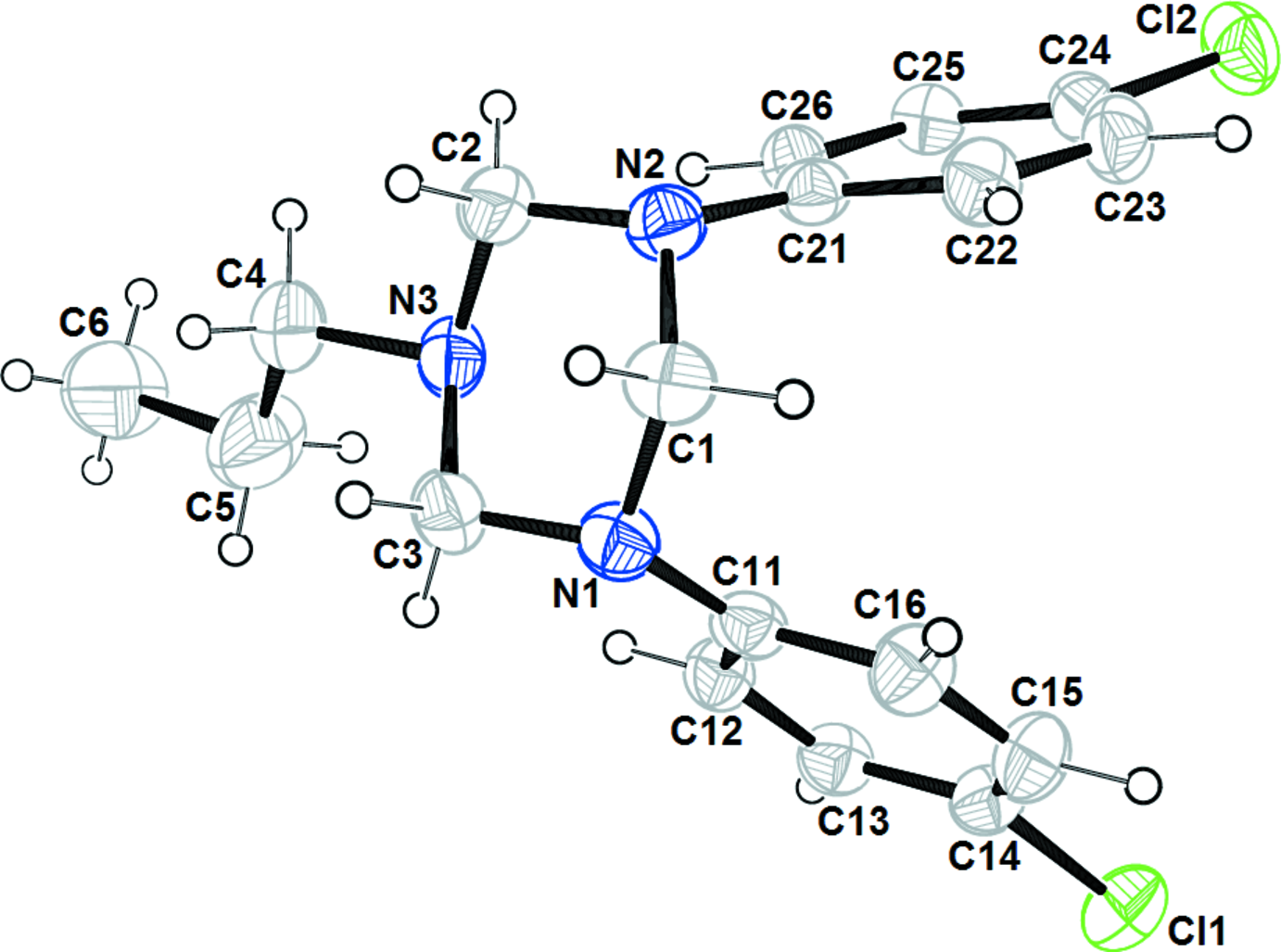

Supplement: Supplementary file 4 [file e-70-o1061-fig1.tif]

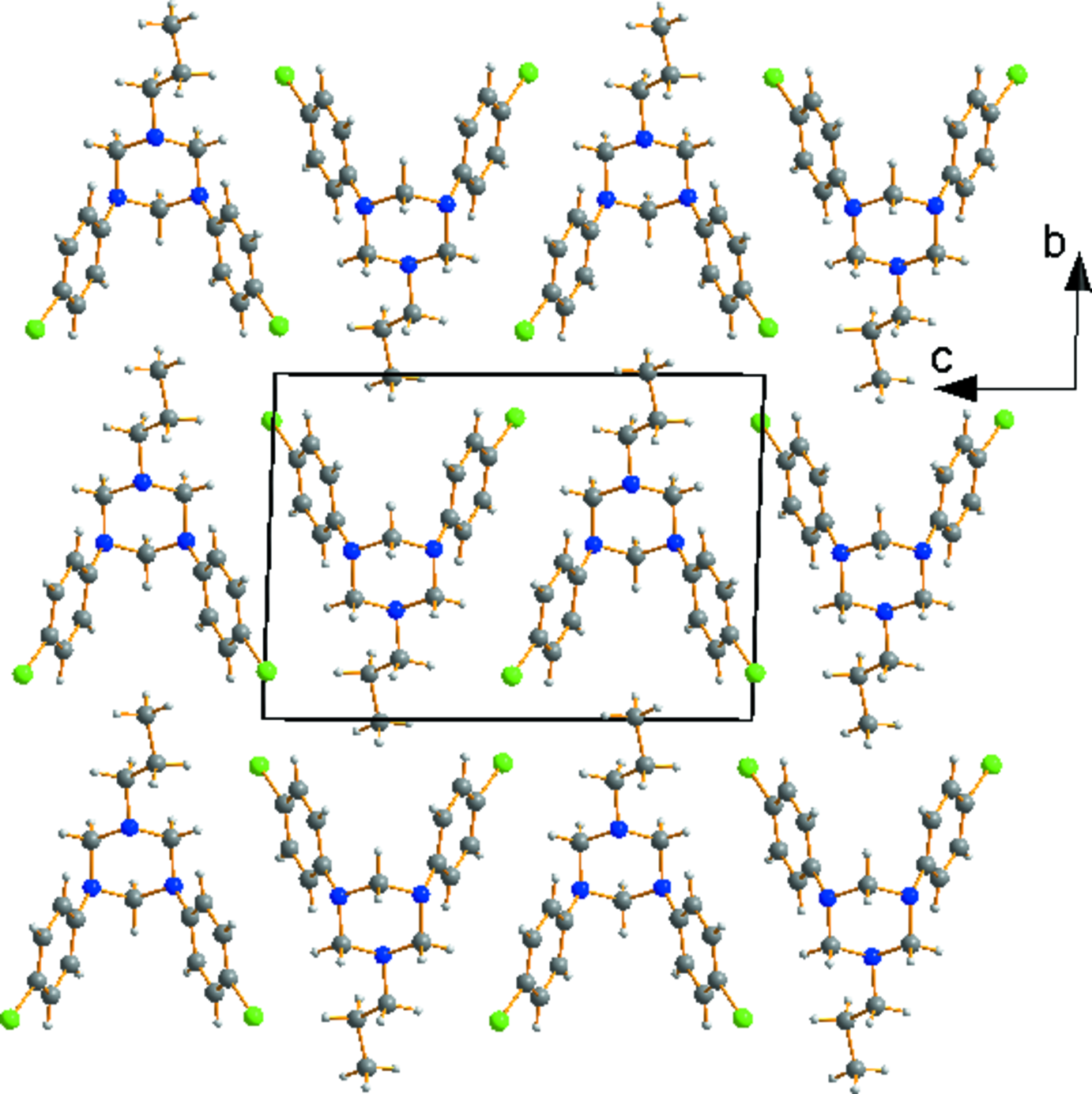

Supplement: Supplementary file 5 [file e-70-o1061-fig2.tif]
